# Outcomes of Acute Coronary Syndrome Patients Who Presented with Cardiogenic Shock versus Patients Who Developed Cardiogenic Shock during Hospitalization

**DOI:** 10.3390/jcm12072603

**Published:** 2023-03-30

**Authors:** Zahi Abu Ghosh, Offer Amir, Shemy Carasso, David Planer, Ronny Alcalai, Mordechai Golomb, Gil Dagan, Eran Kalmanovich, Alex Blatt, Gabby Elbaz-Greener

**Affiliations:** 1 Department of Cardiology, Hadassah Medical Center, The Faculty of Medicine, Hebrew University of Jerusalem, Jerusalem 9190501, Israel; 2 The Azrieli Faculty of Medicine in the Galilee, Bar-Ilan University, Safed 5290002, Israel; 3 The Jesselson Integrated Heart Center, Shaare Zedek Medical Center, Jerusalem 9103102, Israel; 4 Department of Cardiology, Shamir Medical Center, Sackler School of Medicine, Tel-Aviv University, Tel Aviv-Yafo 6997801, Israel; 5 Kaplan Heart Center, Hebrew University, Jerusalem 9190501, Israel

**Keywords:** cardiogenic shock, myocardial infarction, acute coronary syndrome

## Abstract

**Background**: Cardiogenic shock (CS) continues to be a severe and fatal complication of acute coronary syndrome (ACS). CS patients have a high mortality rate despite significant progress in primary reperfusion, the management of heart failure and the expansion of mechanical circulatory support strategies. The present study addressed the clinical characteristics, management, and outcomes of ACS patients complicated with CS. **Methods:** We performed an observational study, using the 2000–2013 Acute Coronary Syndrome Israeli Surveys (ACSIS) database and identified hospitalizations of ACS patients complicated with CS. Patients’ demographics and clinical characteristics, complications and outcomes were evaluated. We assessed the outcomes of ACS patients with CS at arrival (on the day of admission) compared with ACS patients who arrived without CS and developed CS during hospitalization. **Results:** The cohort included 13,434 patients with ACS diagnoses during the study period. Of these, 4.2% were complicated with CS; 224 patients were admitted with both ACS and CS; while 341 ACS patients developed CS only during the hospitalization period. The latter patients had significantly higher rates of MACEs compared with the group of ACS patients who presented with CS at arrival (73% vs. 51%; *p* < 0.0001). Similarly, the rates of in-hospital mortality (55% vs. 36%; *p* < 0.0001), 30-day mortality (64% vs. 50%; *p* = 0.0013) and 1-year mortality (73% vs. 59%; *p* = 0.0016) were higher in ACS patients who developed CS during hospitalization vs. ACS patients with CS at admission. There was a significant decrease in 1-year mortality trends during the 13 years of this study presented in ACS patients from both groups. **Conclusions:** Patients who developed CS during hospitalization had higher mortality and MACE rates compared with those who presented with CS at arrival. Further studies should focus on this subgroup of high-risk patients.

## 1. Introduction

Cardiogenic shock (CS) remains a serious and fatal complication of acute coronary syndrome (ACS), which is associated with high mortality rates [1]. Despite advances in timely reperfusion, and several therapeutic options and treatments for heart failure (HF), high mortality rates are observed [1,2]. Early revascularization, predominantly via percutaneous coronary intervention (PCI) to restore blood flow to the culprit coronary artery, can reduce the mortality associated with CS [3,4,5].

Hospitalizations attributed to CS have tripled in the period from 2004 to 2018 according to a registry from the United States National Inpatient Sample (NIS) [6]. Among these are patients with acute myocardial infarction (MI) with CS [6], while several studies have shown a decreasing trend in mortality among patients with CS [6,7,8,9,10]. This reduction in mortality may be attributed to the expansion of mechanical circulatory support (MCS) strategies, timely revascularization, and advances in intensive care therapy [7,8,9,10].

There are limited data in the literature regarding differences in mortality rates and MACEs among patients who presented with ACS and CS on the day of hospital admission compared with ACS patients who developed CS only during the hospitalization period.

The current observational study evaluated the clinical characteristics, treatment and mortality among patients with ACS and CS at arrival versus ACS patients who developed CS during hospitalization after the first day of admission, all of whom were enrolled in the Acute Coronary Syndrome Israeli Surveys (ACSIS).

## 2. Methods

### 2.1. Data Collection and Study Population

The ACSIS is a national survey that has been conducted in Israel since 1992. The details of these nationwide registries have been previously reported [11,12,13]. Briefly, ACSIS is conducted during a period of two months, once every two years. Data are prospectively collected from all patients who are admitted and eventually discharged with a diagnosis corresponding to the ACS spectrum in each of the 25 coronary care units and cardiology wards operating in Israel. Demographic and clinical data are recorded on pre-specified forms for all these patients. The discharge diagnoses are recorded as determined by the attending physicians based on clinical, electrocardiographic and biomarker criteria. In-hospital and 30-day outcome data are ascertained via a hospital chart review, telephone contact, and clinical follow-up data. Patient management is at the discretion of the attending physicians at each center. Mortality data during hospitalization, at 30-days and one-year post-hospitalization are determined for all patients from hospital charts and by matching the identification numbers of patients with the Israeli National Population Registry. All parameters captured in the registry are defined in the protocol.

The current study population comprises all patients included in ACSIS between 2000 and 2013 who were admitted with ACS and presented with evidence of cardiogenic shock at admission, defined as a Killip score of 4 at admission, and patients with ACS who developed CS during hospitalization (a Killip score of 4 describes individuals in cardiogenic shock or hypotension measured as an SBP below 90 and with evidence of low cardiac output: oliguria, cyanosis or impaired mental status) [14,15]. Definitions, outcome comparisons and trend calculations were made using data from all seven surveys by dichotomizing all patients in this study.

### 2.2. Outcomes

The outcome measures of the present study included the following: in-hospital mortality, 7-day mortality, 30-day mortality, 1-year all-cause mortality and 30-day major adverse cardiac and cerebral events (MACEs) defined as recurrent myocardial infarction, recurrent ischemia, stent thrombosis, urgent repeat revascularization, or death.

### 2.3. Statistical Analysis

The SAS software (Version 8.2, SAS Institute Inc., Cary, NC, USA) was used for statistical analysis. A chi-square (χ2) test and Wilcoxon rank-sum test were used to compare categorical variables and continuous variables, respectively. The Rao–Scott F-adjusted chi-square test was used to represent differences in baseline characteristic frequencies. The Kruskal–Wallis test was used for the comparison of non-normally distributed continuous variables. All tests were 2-sided, and *p*-values of < 0.05 were considered statistically significant. Piecewise regression analyses were performed to assess temporal trends in 1-year mortality.

## 3. Results

A total of 13,434 ACS patients were enrolled in the ACSIS survey between the years 2000 and 2013, among whom 224 patients presented with CS at arrival, and 341 patients developed CS during hospitalization.

### 3.1. Baseline Characteristics

The clinical characteristics of the patients are presented in Table 1. The mean age of the total cohort was 70.9 ± 13.1 (mean ± SD), without significant changes between the 2 groups. In both groups, the majority of the patients were males (Table 1).

No statistically significant differences between the two groups were observed with regard to baseline characteristics and past medical history, including prior MI and congestive heart failure (CHF). Chronic renal failure (CRF) was more frequent in the group of patients who developed CS during hospitalization compared with the patients who had CS at arrival. There was no difference in the chronic medications between the two groups, excluding insulin, which was highly used by patients who developed CS during hospitalization (Table 1).

### 3.2. Clinical Presentation and Management

The clinical presentations of both CS at arrival and CS during hospitalization are presented in Table 2. Patients with CS during hospitalization had significantly higher rates of typical angina in their clinical presentations compared with those with CS at admission ((220/341) 46.5% vs. (101/224) 45.1% (*p* = 0.0001), respectively) (Table 2). Arrhythmias, syncope or aborted sudden cardiac death at presentation were significantly more prevalent in the CS-at-arrival patients (Table 2).

Both patient groups presented mainly with ST-segment elevation in their initial ECG recordings (73.87% vs. 76.18%; *p* = 0.6), and the rate of diagnosis of non-STEMI was similar among patients from both groups (Table 2). The levels of troponin I were higher in patients who developed CS during hospitalization, and other laboratory results show no significant changes between the groups (Table 2).

Around 70% of the patients from both groups underwent primary PCI without significant differences (68.7% and 67.9%; *p* = 0.88) (Table 3). The rate of coronary artery bypass graft (CABG) surgery was similar in both groups.

Additionally, the rates of mechanical ventilation, direct current shock, and cardiopulmonary resuscitation were all similar in both groups; meanwhile, intra-aortic balloon pump (IABP) use was significantly higher in patients who developed CS at hospitalization (49.1% vs. 35.7%; *p* = 0.001) (Table 3). The patients who developed CS during hospitalization were treated more with clopidogrel (60.7% vs. 45%; *p* = 0.0002), heparin (78.9% vs. 69.2%; *p* = 0.009) and diuretics (68.5% vs. 56.8%; *p* = 0.004) compared with CS-at-admission patients. Treatment rates of aspirin, low-molecular-weight heparin, beta blockers and renin–angiotensin system inhibitors were similar between the two groups (Table 3).

### 3.3. Complications

**Figure 1:**
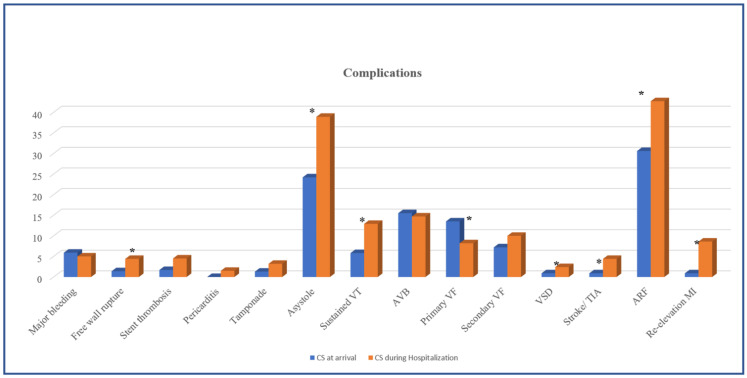
Complications among patients with ACS and CS at arrival versus patients who developed CS during hospitalization. * *p* < 0.05.

Patients with ACS who developed CS during hospitalization had significantly higher rates of several complications compared with CS-at-arrival patients (Figure 1).

**Figure 2:**
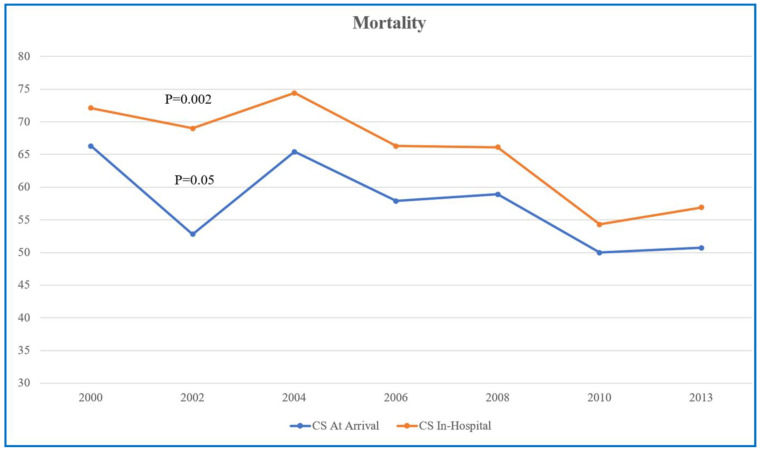
One-year mortality rates in ACS patients with CS at arrival (blue line) and ACS patients who developed CS during hospitalization (red line) between the years 2000 and 2013.

Among these complications were free wall rupture (4.4% vs. 1.4%; *p* = 0.04), ventricular septal defect (VSD) (2.4% vs. 0.9%; *p* = 0.02), re-elevation MI (8.6% vs. 0.9%; *p* = 0.0001), stroke or transient ischemic attack (4.4% vs. 0.9%; *p*= 0.017) and acute renal failure (ARF) (42.7% vs. 30.6%; *p* = 0.003). Furthermore, asystole (38.9% vs. 24.2%; *p* < 0.001) and sustained ventricular tachycardia (VT) (12.9 vs. 5.8%; *p*= 0.006) were significantly higher in ACS patients who developed CS in hospital. In contrast, patients with CS at arrival had higher primary ventricular fibrillation (VF) (13.5% vs. 8.2%; *p* = 0.04) (Figure 2). There were no significant differences in the rates of major bleeding, stent thrombosis, pericarditis, high-degree AV block, secondary VF and cardiac tamponade between the two groups of patients.

### 3.4. Mortality

The rates of all-cause in-hospital mortality, 7-day mortality, 30-day mortality and 1-year mortality were higher in the group of patients with ACS who developed CS during hospitalization compared with the group of patients with ACS who presented with CS at arrival (Table 4). Furthermore, there was a significantly higher rate of MACEs among CS-in-hospital patients compared with CS-at-arrival patients (73% vs. 51.3%; *p* < 0.0001) (Table 4). There was a significant decrease in the trend of 1-year mortality over the years for both patient groups (Figure 2).

## 4. Discussion

The current study evaluated the clinical characteristics, treatment and mortality of 565 patients with ACS and CS. Our unique study compared 224 patients with CS at arrival versus 341 patients who developed CS during hospitalization.

Our findings indicate that there were no significant differences in the baseline characteristics between the group of patients who developed CS during hospitalization and the group of ACS patients who presented with CS at arrival, except in the prevalence of CRF, which was higher in the latter group. Furthermore, patients in both groups presented mainly with ST-segment elevation in their initial ECG recordings and underwent primary PCI at a similar rate.

Interestingly, ACS patients who developed CS during hospitalization had significantly higher mortality rates in all the different time points that were examined—in-hospital mortality, 7-day mortality, 30-day mortality and 1-year mortality—compared with the group of ACS patients who presented with CS at arrival.

This finding could be explained by the fact that patients who were admitted with ACS and CS were most likely prone to reversible causes, and by being treated immediately, their outcomes were better. Moreover, since these patients presented with CS at arrival, they may have received instant intensive treatment and advanced supportive and escalation therapy. In contrast, patients who developed CS during hospitalization had higher rates of mortality, which can be explained by the observational findings that this group had higher mechanical complications such as free wall rupture, VSD and other complications such as sustained VT, stroke and ARF, all of which are associated with high mortality rates according to prior studies [16,17].

Furthermore, our study shows that the development of CS during hospitalization is associated with increased rates of severe complications. The outcomes that revealed high mortality rates among ACS patients with CS from both groups are consistent with previous studies that have similarly shown a very high mortality rate among ACS patients with CS [1,6].

Cardiogenic shock remains a grave complication of ACS associated with high mortality rates despite advances in timely reperfusion and various therapeutic options for heart failure [16,17,18,19]. However, the use of MCS devices demonstrated better hemodynamic and metabolic profiles for patients with CS and can improve symptoms and maybe the survival of patients with advanced HF [20,21,22]. The new ESC guidelines for the diagnosis and treatment of acute and chronic HF published in 2021 recommend the use of short-term MCS as extracorporeal membrane oxygenation (ECMO) or Impella in patients with CS until hemodynamic stabilization and the improvement of end-organ perfusion [23]. Meanwhile, long-term MCS, as a left ventricular assist device (LVAD), can be used when short-term MCS has not led to cardiac recovery or clinical improvement [23].

The current study revealed a significant decrease in the trend of mortality rates between both groups of ACS patients. Why would this occur? There are several factors that may explain this reduction in mortality rates. Early revascularization, predominantly PCI, to restore blood flow to the culprit coronary artery may contribute to the reduction in the mortality associated with CS, which has been demonstrated in previous studies [3,4,5].

Moreover, our study findings indicate that patients who developed CS during hospitalization had a higher prevalence of CRF. CRF is a known risk factor for CS and is associated with higher mortality rates [24,25,26]. This finding highlights the importance of the close monitoring of patients with ACS who have CRF for the development of CS during hospitalization.

More studies should be performed to evaluate whether the advancements in therapeutic options and treatments for heart failure reduce the rate of complications among these patients and decrease their high mortality rates.

Our study has several strengths, including the use of a large, nationwide database, prospective data collection and a long follow-up period. However, the finding of this study should be considered in the context of several limitations that merit discussion. Our data were retrospectively collected, and we could only use the factors that were captured in the registry. As such, we cannot discount the presence of residual confounding, and several limitations should also be noted. This study was limited to patients from Israel, and the generalizability of the findings to other populations may be limited. Furthermore, we do not have details about the timings of the developments of CS during the hospitalizations. Eventually, our current article is based on the data that were collected until 2013, while in the recent era, there has been impressive advancement in the pharmacological and mechanical supportive treatment of ACS patients with CS. However, in our next article dealing with a similar issue, we will describe whether there is a significant impact on the mortality rate in and outcomes of these groups of patients utilizing more recent data from the recent decade.

## 5. Conclusions

Our study suggests that the development of CS among ACS patients during hospitalization is associated with increased mortality rates compared with ACS patients who present with CS at arrival to hospital. There has been a decreasing trend of mortality among ACS patients with CS over the years. Our current study highlighted the group of patients who developed shock during hospitalization with high mortality rates. Further studies should focus on these patients to identify their risk factors, clinical characteristics and complications, and whether the advancement in treatment options contributes to improving their survival rate.

## Figures and Tables

**Table 1:** Demographics and clinical characteristics of patients with ACS and CS at arrival versus patients who developed CS during hospitalization.

Variable *n*, (%)	CS at Arrival (N = 224)	CS during Hospitalization (N = 341)	*p*-Value
Gender (female)	67 (29.9)	118 (34.6)	0.24
Mean age ± SD	69.7 ± 13.10	71.67 ± 12.84	0.25
Diabetes mellitus	78 (35.1)	142 (41.9)	0.15
Hypertension	128 (57.9)	339 (65.8)	0.06
Dyslipidemia	104 (47.9)	180 (53.7)	0.18
Mean body mass index (kg/m^2^)	27.24	28.37	0.65
Prior myocardial infarction	70 (31.8)	131 (38.8)	0.1
Prior percutaneous coronary intervention	38 (17.3)	66 (19.5)	0.5
Prior coronary artery bypass graft	14 (6.3)	31 (9.1)	0.23
Prior stroke/transient ischemic attack	31 (14)	38 (14.2)	0.95
Congestive heart failure	40 (18.2)	58 (17.2)	0.76
Chronic renal failure	32 (14.4)	74 (21.8)	0.03
Peripheral vascular disease	26 (11.8)	56 (16.5)	0.13
Chronic treatment			
Aspirin	80 (47.6)	139 (49.8)	0.65
Clopidogrel	7 (4.1)	24 (8.6)	0.06
Anti-coagulation	9 (5.3)	14 (5)	0.88
Renin angiotensin system inhibitors	61 (34.7)	122 (43.4)	0.06
Beta blockers	67 (39.2)	100 (35.8)	0.47
Calcium channel blockers	32 (19)	50 (18.1)	0.79
Nitrates	28 (16.6)	53 (19.1)	0.5
Statins	56 (33.3)	117 (42.1)	0.06
Insulin	7 (4)	27 (9.7)	0.03

**Table 2:** Clinical presentations and blood lab tests of patients with ACS and CS at arrival versus patients who developed CS during hospitalization.

Variable	CS at Arrival (N = 224)	CS during Hospitalization (N = 341)	*p*-Value
Presentation at arrival *n*, (%)			
Typical angina	101 (45.1)	220 (64.5)	0.0001
Angina > 24 h before admission	54 (24.9)	124 (36.7)	0.003
Atypical angina	19 (10.1)	48 (15.8)	0.07
Dyspnea	82 (36.6)	107 (31.4)	0.12
Sudden cardiac death	52 (23.2)	37 (10.9)	0.0001
Arrythmia	33 (14.7)	25 (7.3)	0.004
Non-ST-segment elevation MI	52 (26.13)	76 (23.82)	0.6
ST-segment elevation MI	147 (73.87)	243 (76.18)	0.06
Hospitalization units			0.003
Intensive cardiac units	192 (85.7)	269 (78.8)	
Internal medicine and other	31 (13.8)	70 (20.5)	
Blood lab tests at arrival			
CK (IU/L) peak value	2573 ± 3428	2374 ± 2559	0.27
Troponin I (ng/mL)	33.20 ± 61.17	41.76 ± 69.7	0.04
Creatinine (mg/dL)	1.76 ± 1.13	1.97 ± 1.39	0.20
Glucose (mg/dL)	246 ± 132.8	225.5 ± 130	0.07
Hemoglobin (g/dL)	12.6 ± 2.2	12.6 ± 2.28	0.83
Total cholesterol (mg/dL)	157 ± 50	172 ± 53.7	0.006
Low-density lipoprotein (mg/dL)	93.6 ± 40.2	100.3 ± 42.5	0.2

**Table 3:** Management of patients with ACS and CS at arrival versus patients who developed CS during hospitalization.

Variable %	CS at Arrival (N = 224)	CS during Hospitalization (N = 341)	*p*-Value
Primary PCI	68.7	67.9	0.88
Total PCI	87	74	0.007
Thrombolysis	10.4	23.2	0.005
Coronary angiography	90.1	92.1	0.06
CABG	5.8	4.1	0.35
CPR	40.7	47.4	0.12
DC Shock	23.1	28	0.19
Mechanical ventilation	66.5	66.5	0.99
IABP	35.7	49.1	0.001
Medication at admission			
Aspirin	83.5	87.9	0.13
Clopidogrel	45	60.7	0.0002
Heparin	69.2	78.9	0.009
Low-molecular-weight heparin	30.8	34.3	0.39
Beta blockers	45.7	46.7	0.82
Renin angiotensin system inhibitors	45.3	49	0.39
Nitrates	25.3	39.1	0.001
Statins	45.2	50.4	0.22
Diuretics	56.8	68.5	0.004
Digoxin	9.5	11	0.56
Glycoprotein IIb/IIIa inhibitors	25.6	25.1	0.89

CABG = coronary artery bypass grafting; CPR = cardiopulmonary resuscitation; DC = direct current cardioversion; IABP = intra-aortic balloon pump; PCI = percutaneous coronary intervention.

**Table 4:** Patient outcomes of patients with ACS and CS at arrival versus patients who developed CS during hospitalization.

Variable	CS at Arrival (N = 224)	CS during Hospitalization (N = 341)	*p*-Value
In-hospital mortality	36.3	55.1	<0.001
7-day mortality	37.7	57.1	0.004
30-day mortality	50.2	64.1	0.001
1-year mortality	59.3	72.9	0.002
MACEs	51.3	73.0	<0.001

MACEs: major adverse cardiac and cerebral events (defined as recurrent myocardial infarction, recurrent ischemia, stent thrombosis, urgent repeat revascularization or death).

## Data Availability

Data are available: gabby@hadassah.org.il.
